# Transcriptome wide SSR discovery cross-taxa transferability and development of marker database for studying genetic diversity population structure of *Lilium* species

**DOI:** 10.1038/s41598-020-75553-0

**Published:** 2020-10-29

**Authors:** Manosh Kumar Biswas, Mita Bagchi, Ujjal Kumar Nath, Dhiman Biswas, Sathishkumar Natarajan, Denison Michael Immanuel Jesse, Jong-In Park, Ill-Sup Nou

**Affiliations:** 1 Department of Horticulture, Sunchon National University, 255 Jungang-ro, Suncheon, Jeonnam, 57922 South Korea; 2 Department of Genetics and Genome Biology, University of Leicester, Leicester, LE1 7RH UK; 3 Department of Genetics and Plant Breeding, Bangladesh Agricultural University, Mymensingh, 2202 Bangladesh; 4 Department of Computer Science and Engineering, Maulana Abul Kalam Azad University of Technology, Kolkata, West Bengal India; 5 Present Address: 3BIGS CO. LTD., 156, Gwanggyo-ro, Yeongtong-gu, Suwon-si, Gyeonggi-do, 16506 South Korea

**Keywords:** Biological techniques, Biotechnology

## Abstract

Lily belongs to family liliaceae, which mainly propagates vegetatively. Therefore, sufficient number of polymorphic, informative, and functional molecular markers are essential for studying a wide range of genetic parameters in *Lilium* species. We attempted to develop, characterize and design SSR (simple sequence repeat) markers using online genetic resources for analyzing genetic diversity and population structure of *Lilium* species. We found di-nucleotide repeat motif were more frequent (4684) within 0.14 gb (giga bases) transcriptome than other repeats, of which was two times higher than tetra-repeat motifs. Frequency of di-(AG/CT), tri-(AGG/CTT), tetra-(AAAT), penta-(AGAGG), and hexa-(AGAGGG) repeats was 34.9%, 7.0%, 0.4%, 0.3%, and 0.2%, respectively. A total of 3607 non-redundant SSR primer pairs was designed based on the sequences of CDS, 5′-UTR and 3′-UTR region covering 34%, 14%, 23%, respectively. Among them, a sub set of primers (245 SSR) was validated using polymerase chain reaction (PCR) amplification, of which 167 primers gave expected PCR amplicon and 101 primers showed polymorphism. Each locus contained 2 to 12 alleles on average 0.82 PIC (polymorphic information content) value. A total of 87 lily accessions was subjected to genetic diversity analysis using polymorphic SSRs and found to separate into seven groups with 0.73 to 0.79 heterozygosity. Our data on large scale SSR based genetic diversity and population structure analysis may help to accelerate the breeding programs of lily through utilizing different genomes, understanding genetics and characterizing germplasm with efficient manner.

## Introduction

Lily (*Lilium* sp.) is an economically important flowering monocots in the genus *Lilium* and family Liliaceae. *Lilium* sp. are originated in Asia, Europe, and North America^1^. The Netherlands are the leading lily producer and exporter in the world^2^, lily also commercially cultivated in France, Chile, USA, Japan, and New Zealand. The *Lilium* genus consists of 100 species and more than 9000 cultivars (International Lily register, https://www.lilyregister.com/). Based on 13 morphological characters the *Lilium* species are taxonomically classified into seven sections; Martagon, Pseudolirium, *Lilium* (Liriotypus), Archelirion, Sinomartagon, Leucolirion, and Oxypetalum^3,4^. All cultivars from these sections were further categorized into three main groups: Longiflorum (L), Asiatic (A), and Oriental (O) hybrids^5^. Longiflorum hybrids derived from intra- or inter-specific hybridization between *L. formosanum* and Leucolirion section^6^, whereas Asiatic hybrids originated from interspecies crosses among 12 species of the Sinomartagon section^7^ and Oriental hybrids derived from hybridization among five species of the Archelirion section^8^. There are many obstacles in the conventional breeding of lily species because of long juvenile phase, self-incompatibility, infertility, pre- or post-hybridization barriers and the traits under quantitative genetics.

Molecular marker assisted breeding (MMAB) could be speeded up lily breeding programs as compared to conventional breeding. Several DNA markers, such as RAPD (randomly amplified polymorphic DNA), ISSR (inter simple sequence repeat), AFLP (amplified fragment length polymorphism), DArT (Diversity Arrays Technology), SSR and SNP (Single Nucleotide Polymorphism) have been developed in lily for studying genetic diversity^9–13^, germplasm characterization, identifying hybrids^9,13,14^, mutants^15^, and genetic mapping^16^. Molecular markers RAPD and ISSR are identified as simple in assay but low reproducibility due to limited in identification of allelic variation within same locus, and unable in characterizing heterozygote because of dominant in nature. Therefore, RAPD and ISSRs have not achieved wide attention in lily breeding. RAPD was used as linked markers of partial resistant of *Fusarium* in 150 individuals of a backcross population of Asiatic hybrids^17^. In where, only three RAPD markers were identified as polymorphic and explained 24% of resistant and concluded that RAPD markers had limitation in lily breeding program. Varshney et al.^18^ also failed to detect variation among the micro-propagated *Lililum* species using RAPD markers. Xi et al*.*^15^ suggested that ISSR may be useful in identification of *L. longiflorum* mutants instated of RAPD. However, Yin et al.^19^ failed to identify mutant of oriental hybrid ‘Siberia’ using ISSR markers, but found polymorphism using AFLP markers among the regenerated ′Siberia' at very low frequency (< 1%).

By developing user friendly, cost effective, transferable and easy-assay molecular markers MMAB program could be further speeded up. Many molecular markers have been developed and used in breeding different crop plants including Ensete^20^, Chrysanthemum^21^ Banana^22^; Citrus^23^. Unlikely, there are limited molecular markers available in *Lilium* sp. In addition, population structure and genetic diversity study in *Lilium* sp. are still not well documented. Recent progress in the sequencing technologies coupled with available bio-informatics tools opened up the avenues of rapid marker development for accelerating the MMAB program. This study attempted to develop large scale SSR markers from the lily transcriptome sequences, subsequently a set of SSR marker uploaded in the freely accessible online lily genomic resources (https://lissrdb.enset-project.org/LiSSRHome.html and mirror link http://genomicsres.org/grn-resources.html). A representative portion of SSR markers were validated and utilized to figure out the lily genetic diversity and population structure of Korean core lily collection.

## Results

### Transcriptome wide SSR discovery, categorization and primer modeling

**Figure 1:**
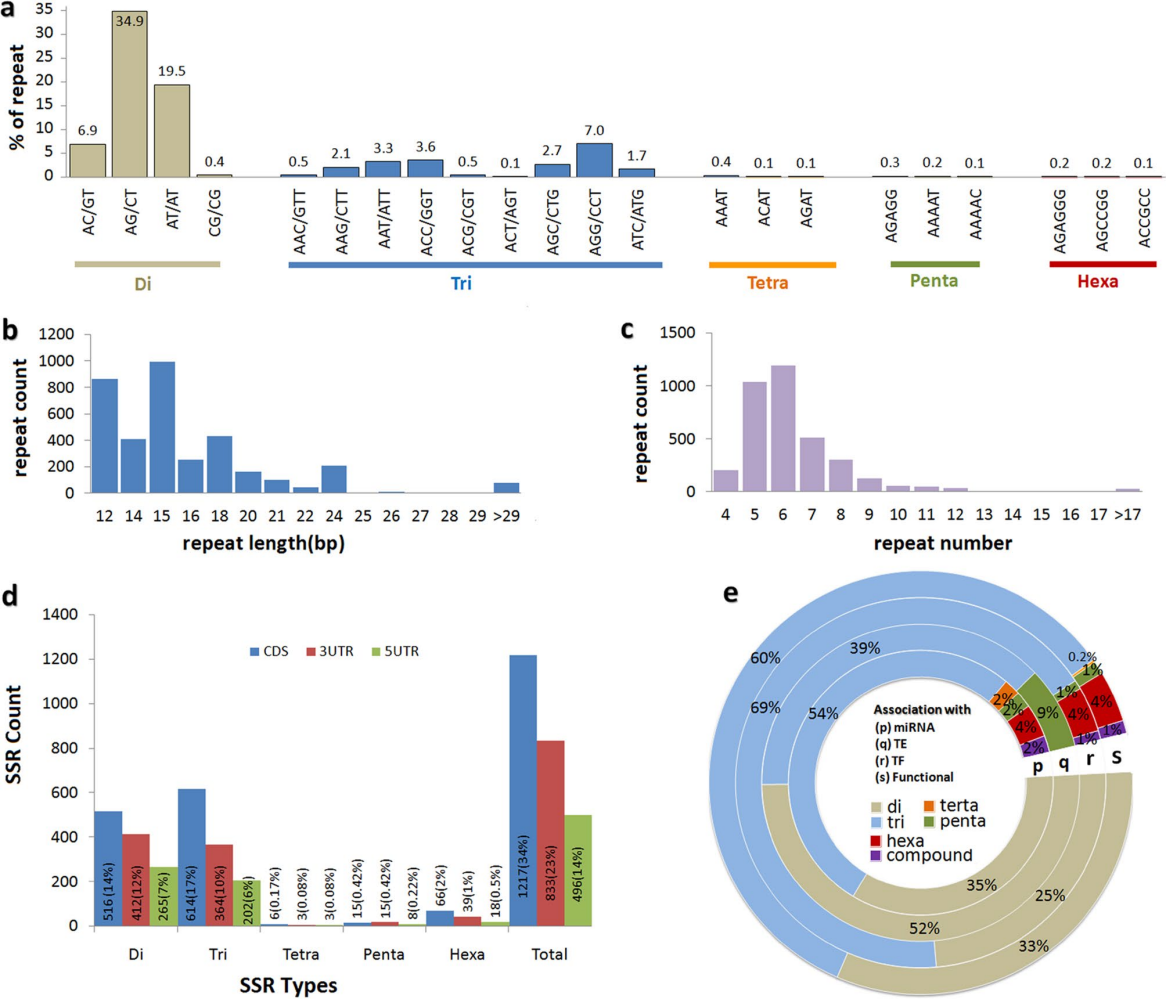
Distribution of SSR repeats in Lily transcriptome sequences: (**a**) frequency distribution of major repeat types, (**b**) distribution of SSR repeats with different length, (**c**) classification of SSR repeats by the number of repeat, (**d**) distribution of the SSR repeat types in different genomic region and (**e**) comparative frequency distribution of SSR types associated with TE (Transposable Element), TF (Transcription Factor) and gene.

A total of 0.11 gb, 0.06 gb and 0.06 gb transcriptome sequences of *L. longiflorum* cv. “Easter”, *L. formolongi* cv. “Sinnapal”, and *L. longiflorum* cv. “White tower”, respectively was assembled (Tables S1, S2) and used for SSR discovery, categorizations and marker development. In total, 7588 SSRs with an average density of 0.054 SSR/kb were picked. Di- and tri-nucleotide repeats found more abundant than other SSR repeats and di-nucleotide repeat was twofold higher than tri-nucleotide repeats. Whereas, terta-, penta- and hexa-repeats found as very low frequency (Table 1). Based on the tract length SSRs were classified into two groups Class I (> 20 bp) and Class II (≤ 20 bp). In this study, Class II SSR found twofold higher than Class I SSR. Over all AT/GC balanced repeats were predominant in lily transcriptome sequences. The most dominant di-nucleotide was AG/CT which accounted 34.9% of the total SSR repeat, followed by AT/AT (19.5%) and AC/GT (6.9%). The most abundant tri-nucleotide repeat was AGG/CCT (7%) followed by ACC/GGT (3.6%) and AAT/ATT (3.3%). Meanwhile AAAT, AGAGG and AGAGGG repeats found as common for tetra-, penta- and hexa-nucleotide, respectively (Fig. 1a). The SSR with 12 to 18 bp in length was more frequent than other types in length (Fig. 1b) and 5 to 7 repeats/SSR loci occurred frequently (Fig. 1c). The SSRs were distributed in the coding regions (CDS) of genome with frequent di- and tri-repeats, accounted 34% (1217) of the total SSR (Fig. 1d). Whereas, only 23% (833) and 14% (496) SSR were located in the 3′- and 5′-UTR regions, respectively.

**Table 1:** Lily SSR mining and categorization statistics.

Item	Count
Total number of sequences examined	216,768
Total size of examined sequences (bp)	139,880,939 (0.14 gb)
Total number of identified SSRs	7588
Number of SSR containing sequences	6966
Number of sequences containing more than 1 SSR	560
Number of SSRs present in compound formation	100
SSR density (SSR/kb)	0.054
Di	4684
Tri	2441
Tretra	73
Penta	149
Hexa	241
ClassII SSR	5309
ClassI SSR	2179
AT rich SSR	2216
GC rich SSR	2115
AT/GC balance SSR	3157

Distribution of lily SSR motifs in the miRNA (microRNA), TEs (transposable elements), TF (transcription factors) and functional protein-coding sequences are illustrated (Fig. 1e, Supplementary Tables S3–S5). Among five types of identified repeats (di- to hexa-), high frequency of tri-repeats found in miRNA, TF, and protein-coding sequences. Whereas, di-repeat was frequent in the TE (Fig. 1e).

To develop transcriptome-wide non-redundant novel SSR markers, 4721 primer pairs were designed from retrieved 7588 SSR, which represents 62% of SSR motif suitable for SSR primer development (Table 2). In-house Perl scripts were used to remove redundant primer sets and one primer was selected from multiple primer sets generated from the same transcript sequences. Finally, 3560 primer pairs were identified as unique and novel lily SSR markers. Of these SSR primers, 1224 (34%) located in the CDS region of the gene. All these markers information are deposited in the freely accessible database (https://lissrdb.enset-project.org/LiSSRHome.html and mirror link https://genomicsres.org/grn-resources.html).

**Table 2:** Lily SSR primer modeling and in silico characterization.

Item	Count	%
Number of SSR primer modeling	4721	62
Number of non-redundant SSR primer	3607	48
One marker from one transcript sequence	3560	47
Number of redundant markers	1161	25
Number of SSR marker in CDS	1224	34
Number of SSR marker in 5 UTR	509	14
Number of SSR marker in 3 UTR	836	23
Number of SSR marker in unknown position	991	28
Number of TE associated SSR	23	1
Number of miRNA associated SSR (no miss match)	48	1
Number of transcription factor related SSR	123	3
In silico polymorphic SSR	645	22
In silico transferable SSR (within in lily)	1139	32
In silico transferable SSR (to other monocot species)	323	9

*TE* transposable elements.

### Functional annotation, miRNA-, TE- and TF-association in SSR

In order to broaden the functional features of SSR marks, we blasted the corresponding sequences of 3560 SSR markers against non-redundant (Nr) protein database (NCBI) database using the BLASTx algorithm with an E-value threshold 1e^−10^. Among them 1260 SSR matched to known proteins in the Nr database. These SSR containing transcriptome matched to 398 plant species in terms of species distribution, the highest number of hit matched to *Elaeis guineensis* (1482; 42%), followed by *Phoenix dactylifera* (1402; 39%), *Musa acuminata* sub sp. malaccensis (1123; 32%), *Nelumbo nucifera* (410; 12%), *Vitis vinifera* (390; 11%) and *Zea mays* (337; 9%) (Supplementary Table S6).

A possible association of the lily SSR with miRNA, TF and TE was determined following the methods describe by Biswas et al.^22^. The associations of SSR loci were determined by BLASTX search of flanking regions against known miRNA, TF and TE libraries. The flanking sequences of non-redundant SSR markers were searched and picked against the custom libraries using a threshold of 65% identity and e-value (< e^−10^). A total of 48, 23 and 123 SSR was associated with the miRNA, TF and TE, respectively (Table 2). Among the TE-associated SSR, 9 SSR had association with LTR/Copia-like TEs followed by 5 SSR with LINE/L1-like, 3 SSR with LTR/Gypsy-like (Supplementary Table S4). We identified 17 SSR associated with bHLH (basic helix loop helix) TF family followed by 10 SSR associated with MYB, 7 SSR associated with C2H2, and 5 SSR associated with G2-Like. Whereas, all 123 TF associated SSR were distributed in 53 TF families (Supplementary Table S5).

### In silico transferability, polymorphism and comparative mapping of SSR

**Figure 2:**
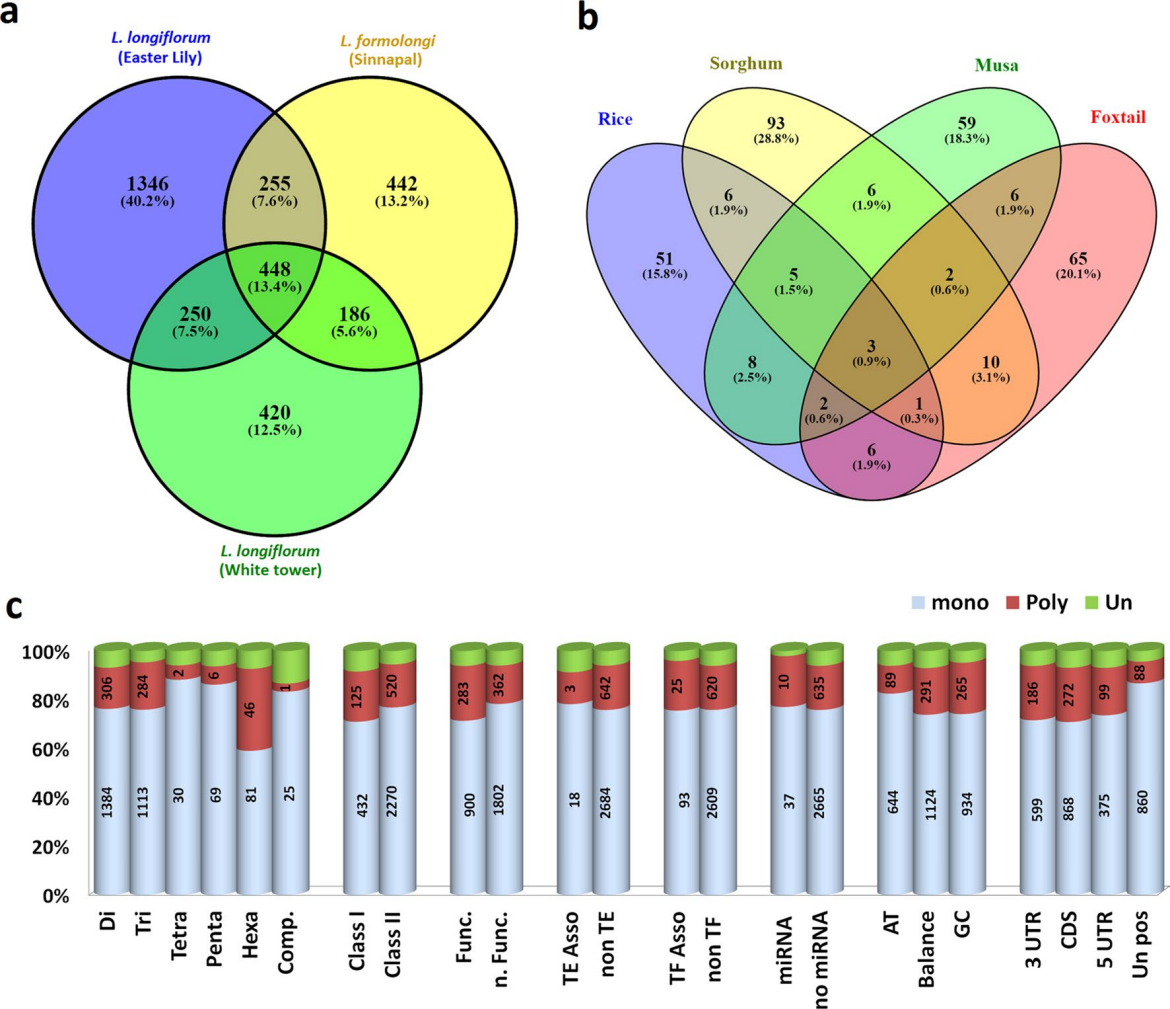
In silico transferability and polymorphism: (**a**) Venn diagram represent the unique and shared SSR markers in three transcriptome dataset, (**b**) transferability of lily SSR markers to other plant species viz. Musa, Foxtail, Rice and Sorghum are represented in this Venn diagram and (**c**) distribution of polymorphic marker among the different types of SSR markers based on in slico analysis results.

In silico validation, transferability and polymorphism analysis of SSR markers were done by using e-PCR approach. All the primers were amplified in silico using three transcriptome data sets separately (*L. longiflorum* cv. “Easter”, *L. formolongi* cv. “Sinnapal”, and *L. longiflorum* cv. “white tower” transcriptome data). Afterward, common primer sets among and between the transcriptome data sets were extracted by using a perl script and visualized in Venn diagram (Fig. 2a) and found 448 SSR (13.8%) common among three data sets which could be transferred among the three lily species. Interestingly, only 255 SSR (7.6%) was common between “Easter lily” and “Sinnapal”, 186 SSR (5.6%) was common between “Sinnapal” and “white tower”, and 250 SSR (7.5%) was common between “Easter lily” and “white tower”. To infer the transferability of the developed SSR to other plant species, the e-PCR analysis was extended to rice, sorghum, banana and foxtail and found 28.8%, 20.1%, 18.3%, and 15.8% of the total SSRs showed transferability to the sorghum, foxtail, banana, and rice, respectively (Fig. 2b). Over all 645 (22.2%) SSRs showed polymorphism among three lily species based in ePCR amplicon (Fig. 2c). Among polymorphic SSRs, SSR with hexa-repeats was highly polymorphic than other SSR marker type. Class I type SSR makers had higher polymorphism compared to class II type SSR (Fig. 2c). Moreover, GC-rich SSR showed higher polymorphism than AT-rich SSR.

### Development of SSR marker database for lily

**Figure 3:**
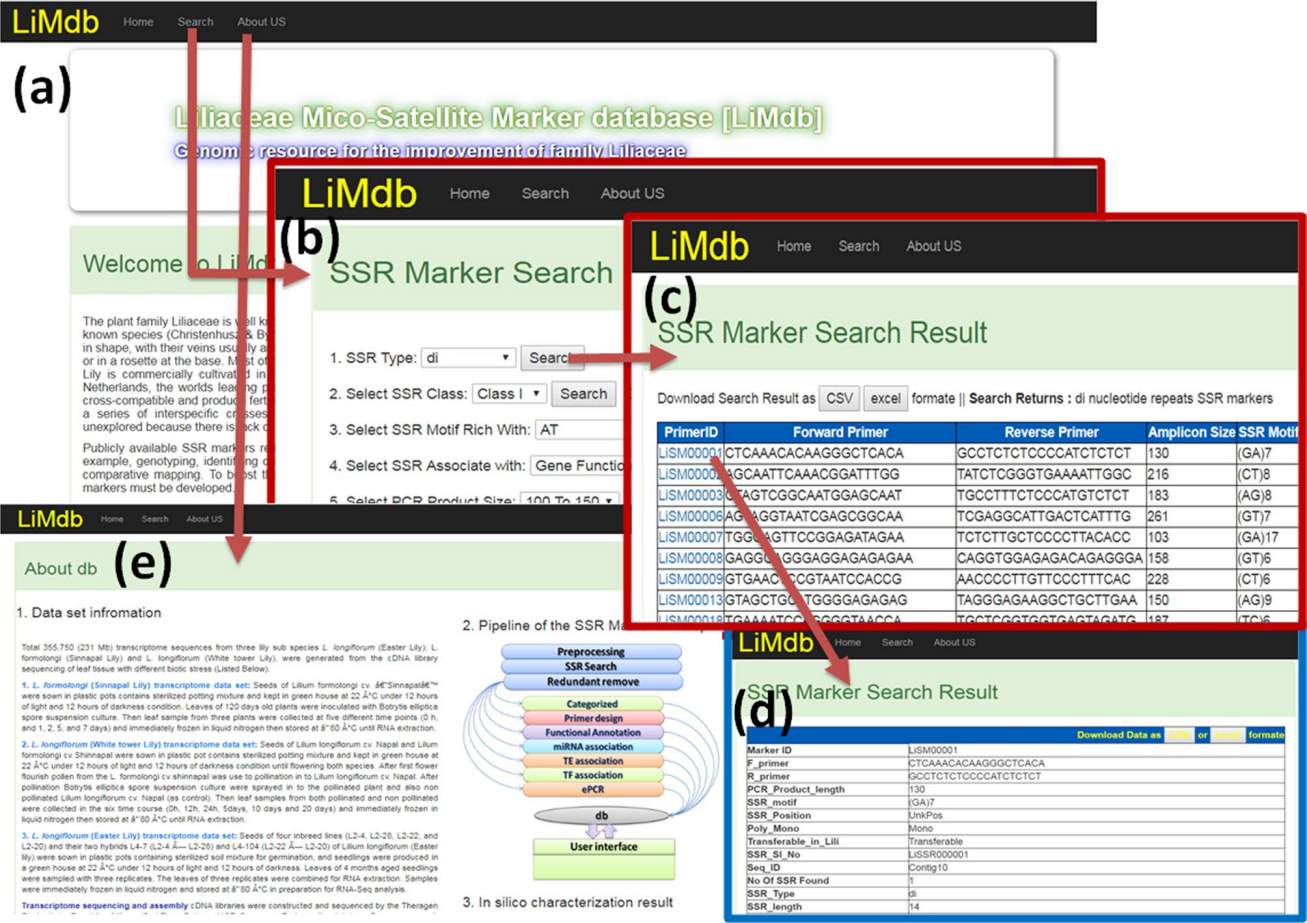
Screenshot of the LiSSRdb showing interface and various utility. (**a**–**d**) Home page SSR search and result display pages and (**e**) details information of the database.

In order to extent the utility of newly developed lily SSR markers, we constructed a user friendly, free accessible online molecular marker database, named LiMdb and available at https://lissrdb.enset-project.org/LiSSRHome.html (mirror link https://genomicsres.org/grn-resources.html). LiMdb consisted three main html pages viz. home, search, and about us (Fig. 3). Home page displays brief introduction of the *Lilium* species, summary of the SSR data and important links of the lily resources. Search page designed with graphical user interface (Fig. 3b). In this page user can search SSR markers by choosing their desired SSR with ten options of different search criteria. Search will be returned back with the SSR marker information according to search criteria in a list as tabular format including the primer ID, forward primer, reverse primer, amplicon size, SSR motif, SSR position, poly-/mono-morphic and transferability information. By clicking on primer ID, detailed information of each SSR marker could be gathered. A single file either CSV or XLS format of desired SSR could be easily downloaded by clicking download button appeared in the page of search result.

### PCR validation, marker potentiality by PIC calculation

Prior to PCR validation, the SSR markers were filtered based on six criteria listed in Supplementary Table S7. We excluded monomorphic (ePCR based) markers, which were out of CDS or UTR region and had no association with miRNA, TF and TE, and also had unknown function in blast2GO analysis. A total 196 SSR markers remained after filtration without any tetra- and penta-nucleotide repeats. In addition, 12 tetra- repeat and 37 penta- repeat markers were randomly picked for wet-lab validation by PCR (Table 3). PCR was performed on a testing panel of six lily accessions included two from Longiflorum, two from Asiatic, one from Oriental, and one out group species (listed in Supplementary Table S8). Among 245 SSR markers, 230 SSR produced PCR amplicon and from there 167 SSR had expected PCR amplicon with product size 100 to 280 bp. Over all 101 (41%) of the tested SSR markers showed polymorphism in six lily accessions. A total 835 alleles was counted from 167 polymorphic SSR with an average 5 alleles/locus (Supplementary Table S9). Most of the SSR amplicon showed 2 alleles/locus. The polymorphism information content (PIC) value was varied from 0.47 to 0.99 with an average of 0.82. The majority of the PIC values were found in the interval from 0.80 to 0.89 (Fig. S1). All these polymorphic SSR markers (101 markers) were used for analyzing genetic diversity and population structure.

**Table 3:** SSR marker selection and PCR validation results.

Types of SSR markers	Before filter	After filter	Select for wet lab	PCR amplify	Specific band	Polymorphic	% polymorphism
Di	1814	69	69	61	40	25	36.23
Tri	1465	114	114	110	92	54	47.36
Tetra	34	0	12	12	5	4	33.33
Penta	80	0	37	35	21	12	32.43
Hexa	137	13	13	12	9	6	46.15
Total	3530	196	245	230	167	101	41.22

### Genetic diversity, phylogenetic relationships and population structure

The genetic diversity and population structure of 87 lily accessions were assessed by using popgene, genealex and structure softwares. In genetic diversity, Asiatic population had the highest PIC (0.77), whereas Oriental population had the lowest PIC (0.58) (Supplementary Table S10). The expected heterozygosity ranged from 0.73 to 0.79 among four populations indicating huge genetic diversity existed among the studied lily germplasm. Pair-wise Nei’s genetic distance ranged from 0.49 to 0.75 for four populations in all possible pairs. The highest genetic distance found between out-group and *Longiforum* population, whereas the smallest distance noted between Asiatic and out-group population (Supplementary Table S11). Pair-wise estimation of significant (*p* < 0.001) Fst among four populations showed high scale of segregation. The highest Fst-value observed between *Longiforum* and out-group, and the lowest between Asiatic and out-group (Supplementary Table S12). Analysis of molecular variance (AMOVA) showed 10% variation among the populations, whereas 90% variation existed within the population, which contributed to make genetic distances of the genotypes (Supplementary Table S12).

**Figure 4:**
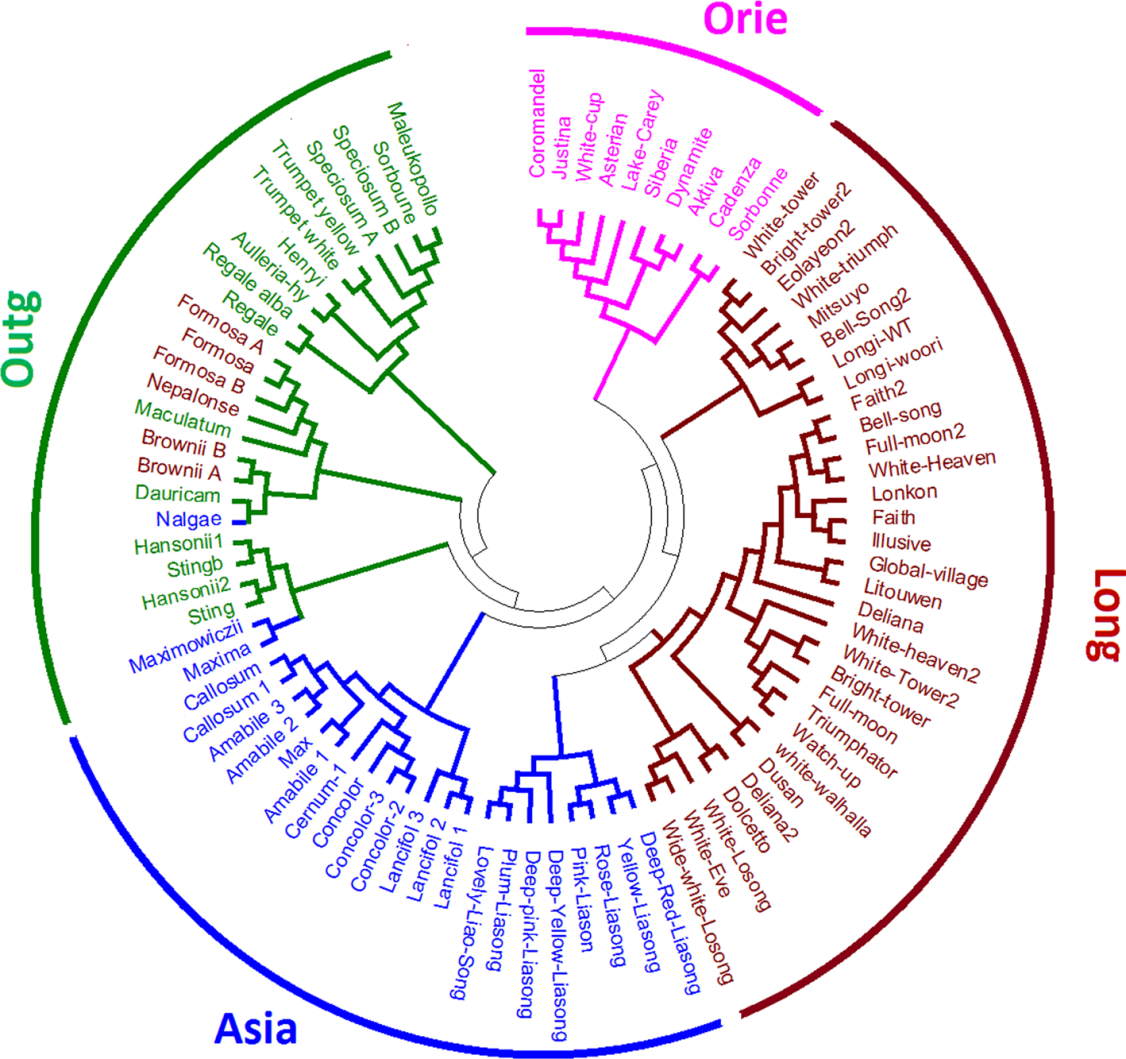
The phylogenetic relationship of 87 lily accession. Neighbor-Joining (NJ) tree constructed by MEGA 6 using 83 SSR markers.

Cluster analysis following neighbor-joined method clearly alienated all 87 lily accessions into different sub-groups based on their genetic background (Fig. 4). All Oriental accessions grouped in a single cluster, Asiatic accessions separated into two major clusters except three individuals (Maxima, Maximowiczii and Nalgae). These three Asiatic accessions shared the clade with out-group accession. Accessions from *Longiflorum* grouped into two different clusters. All out-group accessions grouped into three different clusters among them two clusters shared with *Longiflorum* and Asiatic accessions.

**Figure 5:**
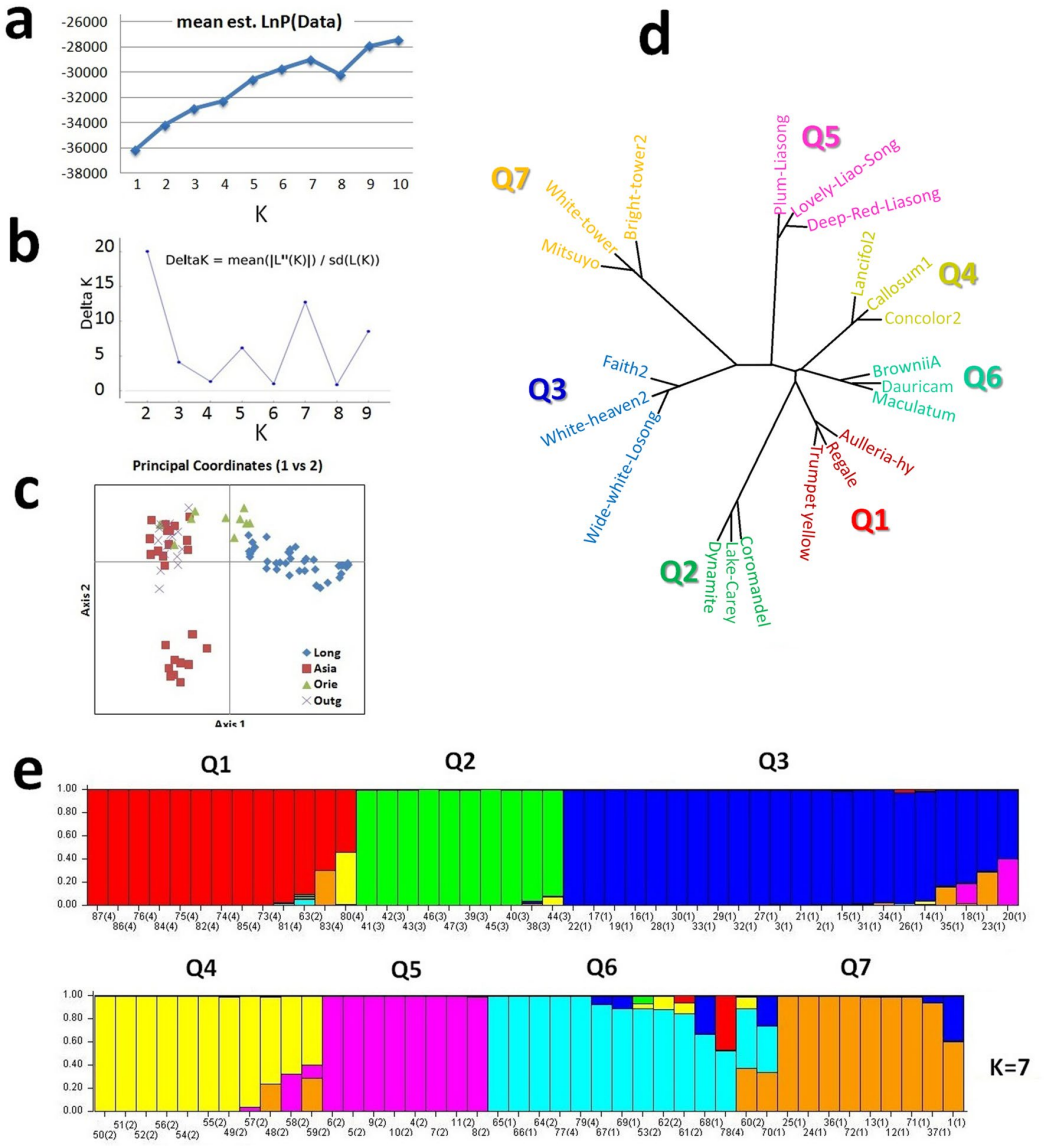
Model-based populations structure analysis: (**a**) plotting the estimated mean value of posterior probability of data LnP (Data)) against the number of populations (K), (**b**) Delta K values for different numbers of populations assumed (K) in the STRUCURE analysis, (**c**) the scatter plot of Principal Coordinates Analysis (PCoA) of the 87 accessions, estimated by GeneAlex software (**d**) phylogenetic tree was constructed using 3 samples from each variety (see accession details in Table S8), where each color represents one cluster matched to the structured population, and (**e**) classification of 87 lily accessions into seven sub populations using STRUCTURE 2.3.1.

Plotting the estimated mean value of posterior probability of data (LnP(Data)) (Fig. 5a) against the number of populations (K) showed gradual growing up the curve from the K values 1 to 7 and thereafter inflated, indicates 87 lily accessions have possibilities to distribute into seven subgroups. However, the delta K value decreased rapidly after K = 2 and showed unstable up and down movement, and further elevated at K = 7 (Fig. 5b). Therefore, based on the LnP (Data) and delta K values whole panel of the lily accessions were subdivided into seven subpopulations (Fig. 5d). Subpopulation Q1 included 13 accessions, of which 12 accessions from out-group and 1 from Asiatic group. All 10 Oriental accessions included into subpopulation Q2. A total 22 accessions clustered into Q3 representing the largest cluster belongs to *Longiflorum* population. Subpopulation Q4 and Q5 contained 11 and 8 accessions, respectively, which belong to Asiatic. Whereas, most of the hybrid genotypes concentrated in the Q6 (Fig. 5e). The phylogenetic relations of these seven subpopulations showed that Q1 and Q2 placed closer than any other subpopulations and Q5 placed as the most distant group. In PCA analysis, *Longiflorum* accessions were more scattered than others, and Asiatic accessions clearly spotted in two different groups (Fig. 5c).

## Discussion

Micro-satellite (SSR) markers are widely used in plant breeding for crop improvement, but its development and utility still limited in many plants species due to limited genome sequences of many species, like *Lilium* species. NGS (Next generation sequencing technology) offers genome- or transcriptome-wide sequencing in reasonable price. These NGS data offer a good resource for developing SSR markers, for example the SSR markers already developed in citrus, banana, and bamboo^22,24–26^. However, transcriptome-wide SSR discovery is still very limited in *Lilium* due to lack of available WGS and transcriptome sequences. We retrieved 195 gb transcriptome data from leaf tissue of three *Lilium* species and subsequently used them for SSR markers mining and development. The SSR retrieving frequencies are comparatively higher in di-cotyledons species than in monocots^22^. In our study, the frequency of SSR retrieving was 0.054 SSR/kb (or 1 SSR per 18 kb), which is much lower than the SSR frequency retrieved in other plant species; especially di-cotyledons; Arabidopsis (1/13.83 kb)^27^ and *Citrus. sinensis* (1/8kbp)^23^. The low frequency of SSRs in *Lilium* transcriptome might resultant to use different criteria in SSR search, software, data types, and species properties^28^. We found abundance of di-nucleotide repeats in lily transcriptome consistent with the previously reported EST-SSR in *Lilium* “Oriental type” hybrid ‘Sorbonne’^13,29^. Moreover, di-nucleotide repeats are dominant in most monocotyledons^30,31^ and some di-cotyledons^32,33^ and the majority of di-cotyledon species have more di-nucleotides than tri-nucleotides^34–36^. We found majority of the di-nucleotide repeats like AG/CT (34.9%) agreed with dominant di-nucleotide repeats reported in *Lilium* “Oriental hybrid” ‘Sorbonne’ (AG/CT, 33.8%)^13,29^.

Previously reported many EST-derived SSR had insufficient flanking regions and low quality of nucleotide composition, therefore failed to use efficiently in plant breeding^37,38^. We also recorded 38% of the lily-SSRs loci found inappropriate in primer development, which is higher than previous reports in citrus^24,33,37^. Percent of designed primers from the transcriptome sequences was less in number due to 100 to 1000 bp transcriptome sequence lengths and exclusion of the designed SSR motifs located in the beginning or end of the transcriptome sequences by the primer design tools due to limited flanking sequence for picking primers with set standard criteria. In approaching pipeline for SSR marker development, primer redundancy was the big concern, because existing SSR mining tools almost unable to optimize primer redundancy. Therefore, significant amount of primer redundancy were encountered in many previous SSR marker development studies^22,23^. The primer redundancy could be raised up to 5–30% during handle a large set of sequences in SSR primer modeling pipeline^22,23^. Almost 25% redundant primers were encountered in this study by applying the pipeline suggested by Biswas et al.^23^.

Distributions and comparison of SSR across different genomes have been reported in many plant species suggested that tri- and hexa-nucleotide are more frequent than other repeats in the protein coding regions (CDS)^23,27,39–42^. We found abundance of tri-nucleotide repeats in lily CDS genomic region. The polymorphic SSRs located in CDS region could modify the coding of protein, whereas SSRs located in UTRs or introns could affect relative expression of gene. Therefore, variable SSR loci located at different genomic regions ultimately leads to phenotypic variability in different genotypes. For example, Li et al.^43^ and Zhang et al.^44^ confirmed the variations of SSR in UTR regions regulating translation of proteins and mRNA stabilization in Arabidopsis. Additionally, Duval and Hamelin^45^ and Vassileva et al*.*^46^ reported MMR gene phenotypes in human connected to SSRs variability in CDS. In lily, tri-repeat density at CDS may provide the opportunity for validating the influence of SSR in gene regulations.

Functional annotation of SSR loci helps to select candidate gene linked to certain traits. Total 35.39% SSR loci of lily found as functionally annotated through blast against NCBI Nr database, whereas, a large quantity (60%) of SSR markers in tea reported as functionally annotated^47^. Functional association of SSR loci and properties of sequence from SSR are retrieved are highly correlated. For instances, SSR loci derived from EST sequences are associated with gene function than the SSR loci derived from inter genomic regions. Functionally characterized lily SSR markers helps in selection of candidate gene associated with phenotypes, of which also confirmed by functional annotation. Moreover, functionally annotated markers are more effective than anonymous markers in marker-assisted selection, trait association analysis, comparative mapping, transcript base mapping, and evolutionary studies. SSRs associated with TE (transposable elements) have been reported in many plant species including *L. bicolor*^48^, Citrus^23^ and Lepidopterans^49^. We identified the low number of TE-associated SSRs in lily coincide with the report in Musa SSR^22^. This findings may be the attributes of TE, because TE are silenced and the sequences used for SSR mining in this study were concentrated on transcribed region of the lily genome, which might be resulted lower number of TE-associated SSR compared to other type of SSR are retrieved (Table 2). TEs located in transcript regions become tended to silence as a result of degrading compatible condition of transcriptional activation^50^. Most of the cases TE-associated SSR markers are amplified in multiple loci due to the presences of multiple copies of TE in the genome^22,49^. TE-associated SSR loci estimated significantly lower level of heterozygosity, which is the major shortfall of using TE-associated SSR markers in breeding programmes.

We found low frequency of polymorphic SSR in in silico ePCR (645; 22.2%) consistent with the observation of SSR markers derived from EST are relatively less polymorphic than genomic SSR markers because EST sequences are conserved within the species compared to genomic sequences^51^. Transferability of the SSR markers to the closely relative species has expanded the possibility in application of designed markers. Whole genome sequences of the closely relative plant species open up the opportunity in in silico transferability of the developed SSR markers among close relatives. This strategy has been applied in many plant species; like banana and citrus successfully for sorting out the high throughput markers from the large number of SSR marker sets. In this study, we estimated the cross species transferability using in silico approach and huge number of lily SSR markers were transferable to other monocot species; like rice, banana, sorghum and foxtail millet (Fig. 2b). It is assumed that these transferable markers could be useful for comparative evolutionary studies of these species.

It is important to reduce number of SSR markers from proposed pipeline for reducing the labor, time and cost involved in web-lab validation. A total 245 SSR markers considering all 3 transcriptome sequences was selected based on six in silico filtering criteria for PCR validation. We recorded 73% of the tested SSR markers amplified with expected amplicon; among them 41% was polymorphic and yielded the average PIC = 0.82 which is slightly higher than previously reported PIC (0.76) in lily^52^. This difference is expected due to use different groups of lily accessions for primer validation. In this study, our major target was to develop highly polymorphic and cross species transferable SSR markers which are worth in many breeding application. In silico (ePCR) and PCR validation results corroborate transcriptome-wide lily SSR markers could be used for germplasm characterization, genetic diversity analysis, population study and QTL mapping of lily populations.

In order to extend the utility of lily SSR markers, we employed them for estimating phylogenetic relationships for characterizing core collection of lily germplasm. In clustering, the *Longiflorum* lily accession distributed in two major clusters and remaining 6 clustered with out-group. Among these six accessions, three were rooted to *Formosa* and two to *Brownie* accessions indicate all of the accessions may be originated from the same parental line or they may be the duplicated accessions in the germplasm collection. The Oriental accessions clustered in a group separated from *Asiatic* and *Longiflorum* and 22 *Asiatic* accessions grouped into two separate clusters. Whereas, Maximowiczii, Maxima and Nagela cultivar *Asiatic* accession clustered in out-group, which might be derived from interspecies crosses. A separate group for Oriental accession agreed with Du et al*.*^13^ reported that Oriental accessions and its hybrids grouped in a single cluster indicates a distinct group of lily apart from *Asiatic* and *Longiflorum*. The “admixture model” of STRUCTURE is commonly used in population genetics to assess the ancestry and variation among individuals. We noted few of the accessions are hybrid in origin. Lilies are vegetatively propagated crop plants and maintained their genomic purity, but we presume some add mixture in our study.

## Conclusions

Microsatellite are robust molecular markers for many plant breeding applications including identification of germplasm, estimation of genetic diversity, and analysis of population structure in both vegetative and non-vegetative propagated crops. Lilies are propagated vegetatively; their genetic diversity, phylogenetic relationships and population structure remained unclear due to lack of insufficient molecular markers. Breeding improvement of lilies is stayed so far behind than many other plant species. We developed transcriptome wide large number of SSR markers by sketching online tools to search lily SSR markers. In addition, we estimated genetic diversity and studied population structure of South Korean lily germplasm core collection. We report a key set of lily SSR markers for characterizing lily accessions, which will be provided enormous genomic recourses valuable for lily genetics, genomics, and breeding.

## Methods

### Lily transcriptome assembly, SSR mining and marker development

Lily transcriptome sequences generated from three lily inbred lines (one from easter lily, one sinnapal and one from white tower) using Illumina sequencing technology (Table S1). Raw reads were filtered and assembled with Trinity following default parameters. All the uni-genes from three data sets were pooled and reassembled into non-redundant uni-genes (Table S2) using CAP3 with default parameters (https://mobyle.pasteur.fr/cgi-bin/portal.py#forms::cap3).

In order to SSR mining and high therapeutic primer development, a pipeline called LSAT (Liliaceae SSR Analysis Tool; https://210.110.86.160/Lsat/Lsat.html or http://genomicsres.org/grn-resources.html) integrated with SSR mining programme MISA (https://pgrc.ipk-gatersleben.de/misa/), primer design tools Primer3, e-PCR, and Blast tools were set up. For the automation of this pipeline we proposed several perl based script to link up each tools. In this pipeline, fasta format of uni-genes sequences were invoked as input data file for subsequent process. Entire pipeline was hosted in the Linux server (https://210.110.86.160/Lsat). Preliminary sequences were checked for quality and format, then invoked in a perl script as fasta file by removing ambiguous and low quality sequences. Poly A/T was removed from the vector and the sequences were trimmed to make free from contamination. Cleaned and processed sequences were used for SSR mining by using MISA programme with the parameters: di- ≥ 6 nt, tri- ≥ 5 nt, tetra- ≥ 5 nt, penta- ≥ 4 nt and hexa- ≥ 4 nt. Based on the motif length (class I > 20 nt and class II ≤ 20 nt) and nucleotide base composition of the motif (AT-rich, AT/GC-balanced and GC-rich) the SSRs were categorized by using a perl script. Primer3 software with batch mode version was used to design SSR primers with default parameters. Finally, the redundant primer sets were removed by using another perl script and identified the uni-genes contained more than one SSR primer sets. One representative primer set of a particular uni-gene was picked from multiple sets of primer with similar sequences. Stepwise outline of this pipeline is illustrated in Fig. S2.

### Functional annotation and go enrichment of the uni-genes of SSR

Blast2Go tool was used for assigning the putative function of the developed lily SSR markers. BLASTX search^53^ was performed in NCBI non-redundant protein database with the threshold 1e^-10^ for the SSR containing uni-genes. The putative function was assigned according to BLASTX hits tools of Blast2Go.

### Analysis of SSR associated miRNA, TF and TE

To identify SSR associated with miRNAs, uni-genes were blasted against known mature miRNA sequences in the miRNA Registry Database V20^54^. BLASTN search fixed the threshold − e1000 with word size − w7. SSR containing uni-gene showed 0 nucleotide mismatched with known miRNAs were considered as SSR markers associated with miRNA. A perl script was used to extract the SSR markers associated with miRNA. SSR markers associated with TF were identified by using BLASTX search fixed with cut off e−10 against Plant TF (transcription family) data base version 3.0 (https://planttfdb.cbi.pku.edu.cn/index.php). A perl script was used to filter SSR associated with TF by setting cut-off value as 65% query coverage and 40% identity from the blasted uni-gene. SSR markers associated with TE were identified following the procedure described by Biswas et al*.*^22,23^. A custom TE library created following the methods described by Xu et al*.*^55^ was used for BLASTX search against the uni-genes with SSR fixing the threshold as 65% identity and e-value < e^−10^.

### In silico transferability, polymorphism and comparative mapping of the SSR markers

In silico transferability of the SSRs to other monocots was assessed by e-PCR (online PCR) considering alternate 3 nucleotides miss-matched and gap. The e-PCR amplicons were compared with expected amplicon size for each marker. The markers were identified as polymorphic and monomorphic based on 6 bp variation in amplicon size; the polymorphic (≥ 6 bp) and monomorphic (< 6 bp).

### Development of database for SSR markers

A user-friendly, free access lily SSR) marker database was constructed as web interface designed by using HTML (Hypertext Markup Language) and JAVA script compatible with various browsers like Google Chrome, Mozilla Firefox and Internet Explorer. The PHP (Hypertext Preprocessor) was used to communicate interface and database server. Information regarding SSR primers, their flanking sequences, putative function and corresponding details have been catalogued in the MySqlserver (version 5.0.77). This database is hosted in c-panel hosting server (https://genomicsres.org/grn-resources.html).

### Plant material and DNA preparation

Details of the plant materials used in this study are listed in Supplementary Table S10. Total genomic DNA was extracted from fresh green and young leaves using the Qiagen DNeasy Plant Mini Kit (QIAGEN, Hilden, Germany) following the protocol supplied as user guide inside kit. DNA quantity and quality were estimated by Nanodrop Spectrophotometer (Thermo Scientific, DE, USA) and 1% agarose gel electrophoresis, respectively. Extracted DNA was diluted to 50 ng/µL for wet lab PCR.

### Experimental validation

Genetic potentiality of the SSR primers were tested by PCR amplification using 6 lily accessions representing diverse genomic groups of the core collection of lily germplasm, maintained at three different locations in South Korea (The National Institute of Horticultural and Herbal Science, Korea National College of Agriculture and Fisheries, and Gangwondo Agricultural Research and Extension Services). The polymorphic SSR markers were used for further large scale genotyping and population structure analysis. PCR was carried out with 20 µL of total reaction volume, contained 1 µL (50 ng/µL) of genomic DNA, 2.5 µL 1 × Taq polymerase buffer, 1.5 µL (2 mM) MgCl_2_, 0.5 µL (0.2 mM) dNTPs mix, 1 µL (10 mM) forward and 1 µL (10 mM) reverse primers, 0.25 µL (1U) Taq polymerase enzyme and 12.25 µL DDH_2_O. PCR was performed in a Takara PCR thermal cycler (TAKARA, Osaka, Japan) applying the following profile: 94 °C for 5 min, 35 cycles of 94 °C for 30 s, 56–60 °C (for different SSR based on melting temperature) for 30 s, and 72 °C for 45 s, and the final step at 72 °C for 5 min. Amplicon size and PCR specificity was tested on 1.5% agarose gel electrophoresis. A 100 bp molecular ladder was used to estimate the amplicon size and the DNA bands were visualized by HIQ Blue Mango (20,000 ×) (bioDx, Daejong. Korea) staining. PCR products of selected primer pairs were evaluated in QIAxcel advanced (QIAGEN, Hilden, Germany) automated DNA fragment analyzer for estimating polymorphism.

### Data analysis

The genetic parameters including number of alleles, polymorphic information content (PIC)/locus, expected heterozygosity (He), observed heterozygosity (Ho), pair-wise comparisons of genetic distance among the species^56^, and Fst (genetic differentiation) were estimated by using software PowerMarker version 3.25^57^. Principal coordinate analysis (PCoA) for population differentiation was performed using the dissimilarity matrix data and Molecular Variance (AMOVA) was estimated to assess the variance between populations and among the genotypes within populations using GenAlEx software version 6.5^58^. Phylogenetic trees were constructed using the unrooted neighbor-joining tree method based on shared genetic distance in PowerMarker and displayed by using MEGA 6.0^18^.

STRUCTURE software^59^ compatible with windows 7 following admixture model and a burn-in period length of 10,000 and MCMC iterations 100,000 were used for estimating population structure. Five independent runs were performed for each K (number of population) value counted from K = 1 to K = 10. The best number of K was then selected with delta K method using the Structure Harvester software^60^. Barplot of the Q matrix was drawn by DISTRUCT software^61^. All graph and figures in this study generated and edited by using MS-xls and MS-paint.

## Supplementary information

Supplementary Tables.

Supplementary Figures.

## Data Availability

Data generated in this study are deposited in the online portal with free accessibility (https://lissrdb.enset-project.org/LiSSRHome.html and https://genomicsres.org/grn-resources.html).
