# Supplementary material for: Transcriptome wide SSR discovery cross-taxa transferability and development of marker database for studying genetic diversity population structure of Lilium species
Source: Sci Rep. 2020 Oct 29;10:18621. doi: 10.1038/s41598-020-75553-0 (PMC7596044; doi:10.1038/s41598-020-75553-0)
Supplement: Supplementary file 2 — Supplementary Figures. [file 41598_2020_75553_MOESM2_ESM.docx]

Transcriptome wide SSR discovery cross-taxa transferability and development of marker database for studying genetic diversity population structure of *Lilium* species

Manosh Kumar Biswas^1,2^, Mita Bagchi^1,2^, Ujjal Kumar Nath^1,3^, Dhiman Biswas^4^, Sathishkumar Natarajan^1^, Denison Michael Immanuel Jesse ^1^, Jong-In Park^1^, Ill-Sup Nou^1^*

^1^Department of Horticulture, Sunchon National University, 255 Jungang-ro, Suncheon, Jeonnam, 57922 South Korea.

^2^Department of Genetics and Genome Biology, University of Leicester, Leicester, LE1 7RH, UK

**^3^**Department of Genetics and Plant Breeding, Bangladesh Agricultural University, Mymensingh-2202, Bangladesh

^4^Department of Computer Science and Engineering, Maulana Abul Kalam Azad University of Technology, West Bengal, India.

**
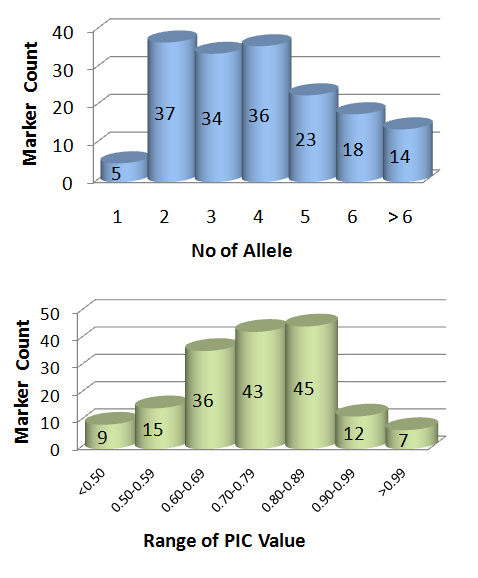
**

**Figure S1.** Distribution of number of allele per locus and PIC value.


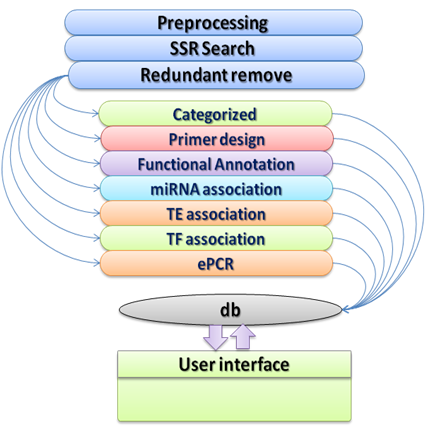


**Figure S2.** Work flow of the lily SSR marker development.
